# Assessing the impact of social determinants of health on rising preterm birth trends in England. A longitudinal ecological study, 2012–17

**DOI:** 10.1093/pubmed/fdag045

**Published:** 2026-06-16

**Authors:** Amy Wilcock, Davara Bennett, David Taylor-Robinson, Philip McHale

**Affiliations:** Division of Population Health, Health Services Research & Primary Care, School of Health Sciences, University of Manchester, Oxford Road, Manchester M13 9PT, UK; Department of Public Health, Policy and Systems, Institute of Population Health, University of Liverpool, Brownlow Street, Liverpool L69 3GF, UK; Department of Public Health, Policy and Systems, Institute of Population Health, University of Liverpool, Brownlow Street, Liverpool L69 3GF, UK; Department of Public Health, Policy and Systems, Institute of Population Health, University of Liverpool, Brownlow Street, Liverpool L69 3GF, UK; Department of Public Health, Policy and Systems, Institute of Population Health, University of Liverpool, Brownlow Street, Liverpool L69 3GF, UK

**Keywords:** Social determinants of health, socioeconomic inequalities, preterm birth

## Abstract

**Background:**

Preterm birth (PTB) is the UK’s leading cause of neonatal morbidity and mortality. There is international evidence of socioeconomic inequalities in PTB. Between 2012 and 2017, the proportion of live births occurring prematurely increased in England. This study explores how changes to major social determinants of health (SDH) may have contributed to recent trends.

**Methods:**

We analysed PTB trends and their correlation with contemporaneous trends in major SDH predictors (socioeconomic status, income, and unemployment) for English Local Authorities. We used both fixed-effects and mixed-effects modelling to estimate the association between SDH and PTB rates between 2012 and 2017.

**Results:**

The gap in PTB inequalities widened over the study period, with the absolute difference in PTB rates between most and least disadvantaged communities rising by 35%. Mixed-effects analysis demonstrated an association between the proportion of children living in low-income households and PTB; for each percentage point increase in child poverty, an estimated additional 0.24 per 1000 births was preterm.

**Conclusion:**

We provide evidence of rising PTB rates in England prior to the pandemic, which disproportionately affected the poorest areas of the country. Policies targeting areas with higher child poverty rates are likely to reduce inequalities in PTB in England.

## Introduction

Preterm birth (PTB) is a leading cause of infant mortality and morbidity in the UK.^1^ Complications of prematurity are multisystemic and include neurodevelopmental abnormalities, chronic lung disease and gastrointestinal disorders.^2^ PTB also presents a high financial burden to the UK economy, with estimated total annual public sector costs of almost £3 billion.^3^

Between 2012 and 2017, the percentage of live births that occurred preterm in England and Wales rose year by year from 7.2% in 2012 to 7.9% in 2017.^4^ Though this figure fell to 7.5% in 2020, the latest data for 2022 indicate that 7.9% of live births were preterm.^5^ Births within the subcategory of ‘moderate preterm’ are fuelling the rise, with ‘very preterm’ and ‘extremely preterm’ births remaining largely static. PTB poses a significant public health threat and the apparent rise, and broadly sustained increase, in the percentage of PTBs in England latterly is concerning. Recently, there have been growing calls from the public and health experts to prioritize maternal and child health outcomes in the UK, leading to increased policy development. In 2022, the first Women’s Health Strategy for England was published by the UK Government to improve the health of women and girls.^6^ An extraordinary 100 000 women and 400 health-related organizations and experts responded to a call for evidence to inform the strategy’s development, reflecting a strong desire for improvement.^7^ In 2024, the Child Health Action Plan was launched to ‘secure the healthiest generation of children ever’.^8^ It is therefore of paramount importance that we urgently adopt robust preterm prevention approaches that address all major contributory factors.

It is widely accepted that social determinants of health (SDH) are powerful influencers of population health and wellbeing. SDH are ‘the conditions in which people are born, grow, work, live and age’.^9^ The Canadian Institute of Advanced Research estimates that the socioeconomic and physical environments collectively contribute to 60% of the health status of the population, with the healthcare system and biology/genetics contributing just 25% and 15%, respectively.^10^ There is consistent evidence demonstrating socioeconomic inequalities in PTB at both the individual and area levels.^11–13^ However, the quantifiable impact of major SDH on recent rising PTB rates in England has not been elucidated. It is vital that we address this clear gap to guide strategic prioritization and resource allocation in PTB prevention.

In this longitudinal study, we investigate trends in PTB between 2012 and 2017 in England, to assess if inequalities were rising over this period, and to elucidate the impact of socioeconomic status (SES), income and employment on PTB rates in England.

## Methods

We undertook a longitudinal ecological analysis of the PTB rates between 2012 and 2017 and their correlation with SES, income, and employment across all lower-tier local authorities (LAs) in England. We used routinely available data from all 333 LAs in England.

### Study variables

#### Primary outcome

The primary outcome measure in this study was PTB rates. Publicly available LA-level crude PTB rates, produced by the Office for National Statistics (ONS), were analysed.^14^ For this indicator, crude PTB rates are defined as the number of preterm live births and stillbirths (gestational age ≥ 24 weeks but < 37 weeks) per 1000 total births (live births and still births).^15^ Each data point within this dataset is presented as a 3-year rolling average. However, to ensure consistent time intervals for data points across the exposures and outcome in this analysis, it will henceforth be referred to as the middle year within this period. For example, the data point for 2012 in this analysis will correspond to the 2011–13 data point within the dataset. As such, there will be overlap between consecutive ‘year’ outcome values.

#### Predictor variables

The Index of Multiple Deprivation 2019 (IMD2019) quintiles were used as an area-level measure of SES. IMD is a time-invariant weighted composite measure of multiple aspects of deprivation for Lower-layer Super Output Areas across England.^16^

As no direct measure of maternal income was routinely available, the percentage of children (<16 years) in low-income families was used as a proxy.^14^^,^^17^ This indicator is defined as those children in families who either receive out-of-work benefits or receive tax credits and have a reported income < 60% of median income, for each LA. To simplify, this will be termed ‘Child Poverty’. Data were available for each yearly interval for the 2012–16 period. A second proxy indicator, affordability of home ownership ratio, was also used to capture income trends.^18^ This is the ratio of median house prices to median gross yearly residence-based earnings. Estimates are available on a single-year basis. To facilitate interpretation, this indicator will be termed ‘Home Unaffordability’.

Finally, to capture employment trends, LA-level model-based unemployment estimates published by the ONS were used.^14^ This indicator was defined as the percentage of economically active persons (≥16 years) not in employment, who had been actively seeking work in the 4 weeks prior to interview and were available to start work in the 2 weeks post-interview. Estimated values were available for each yearly interval, for the 2013–17 period.

### Statistical analysis methods

Firstly, we performed exploratory analysis to examine the apparent rising trend in PTB between 2012 and 2017 across English LAs. This included a comparison of PTB rates across IMD quintiles. We used the Mann–Kendall test to determine if PTB data for this period represented a monotonic time trend and to assess the strength of the relationship.

Data visualization using spaghetti plot analysis revealed limited within-LA variation in our predictor variables, particularly child poverty, over the study period. Therefore, to estimate the effect of ‘unemployment’ and ‘home unaffordability’ on LA PTB rates, we used a two-stage approach, comprising fixed-effects regression followed by linear mixed-effects modelling. Fixed-effects analysis uses only within-LA variation, enabling the estimation of the effect of our independent variables whilst eliminating time-invariant confounders.^19^ Linear mixed-effects models (LMMs) capture between-area and within-area variation and therefore were likely to be informative and improve precision of estimates in the context of limited within-LA variation. For the LMMs, we designated the predictors (IMD, Child Poverty, and Unemployment) and ‘time’ as fixed effects, and ‘LA’ as a random effect. We also accounted for the potential confounders of marital status and maternal age as fixed effects.^20–22^

For ‘child poverty’, we opted to limit analysis to mixed-effects modelling. This was for two reasons: the particularly limited within-LA variation seen during data visualization and concerns about the quality of the child poverty dataset. The HM Revenue and Customs unofficial child poverty estimates used represented the best available LA-level child poverty dataset. However, the accuracy of this dataset has been questioned as its analysis revealed a downward trend in the number of children in low-income families, where the official ‘Households below average income’ data indicated an upward trend for the same period.^23^ Due to the longitudinal within-LA assessment that is performed through fixed-effects analysis, these issues present a significant risk of bias. However, as mixed-effects modelling analyses both within-LA and across-LA variation, we feel we can be more confident in the LMMs for child poverty.

The fixed-effects models and LMMs were calculated using the plm and lme4 packages respectively in R V4.5.1.

### Missing data

Two local authorities (Bournemouth, Christchurch and Poole, and Dorset) were omitted from the data analysis due to geographical boundary changes occurring within the study period. Additionally, the Isles of Scilly and City of London were excluded due to their small size and data suppression. Given that there was minimal missing data (see Supplementary Appendix), listwise deletion was used to manage it.

## Results

### Descriptive analysis

The mean PTB rate for LAs in England was higher in 2017 (80.6 per 1000, 95% CI 79.6–81.6) than it was in 2012 (75.6 per 1000, 95% CI 74.8–76.4). The Mann–Kendall trend test revealed a statistically strong positive trend in the mean PTB rates for England between 2012 and 2017 (*P* = .008).

**Figure 1:**
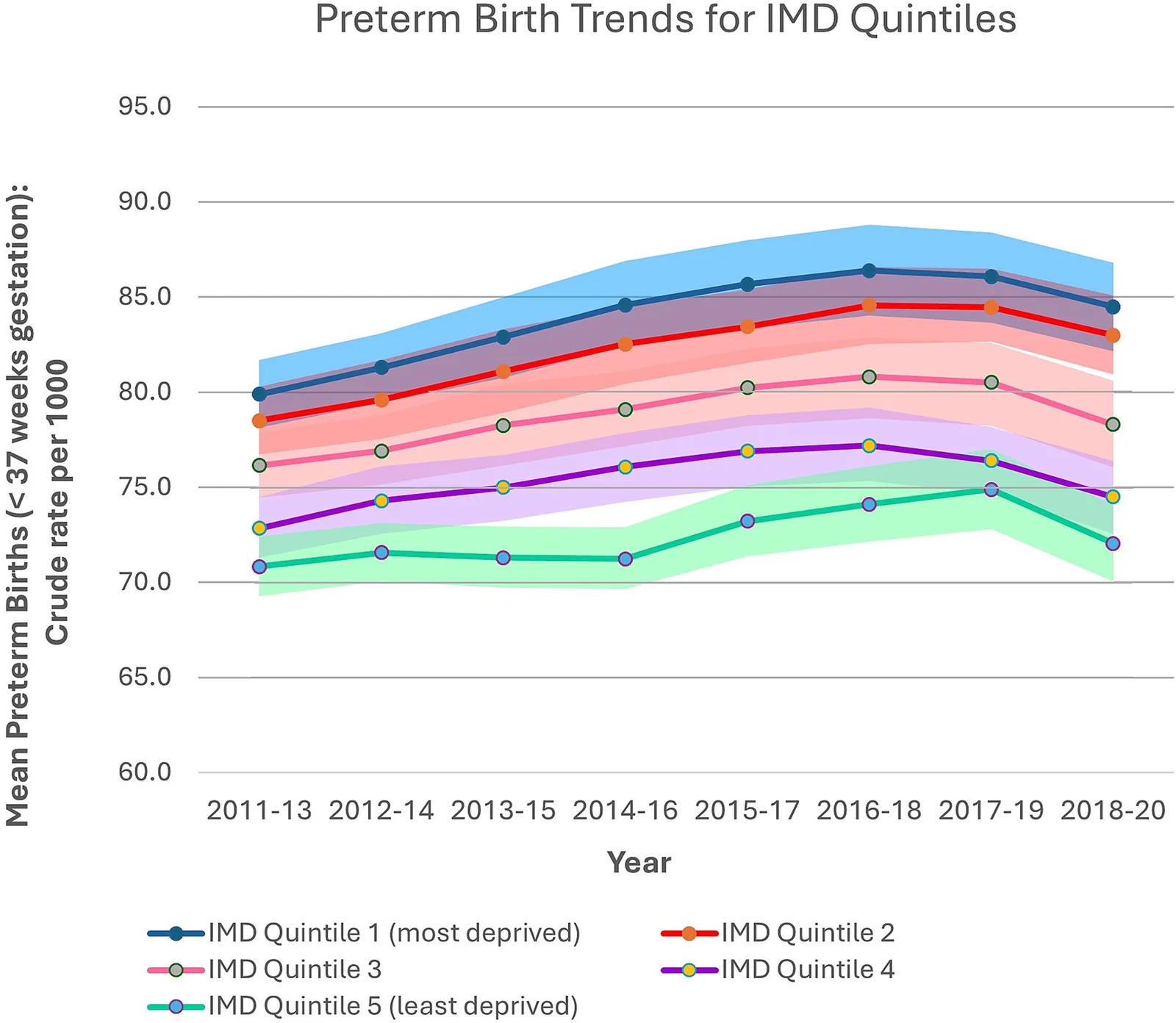
A graph of time trends in mean PTB rates for LAs across England according to IMD quintile classification. Data sourced from OHID (2021).

An IMD average rank was available for 307 LAs. Analysis showed that PTB rates increased between 2012 and 2017 for local authorities across all deprivation quintiles (Fig. 1). However, a socioeconomic gradient in PTB rates was observed, with higher rates for LAs with higher relative deprivation at each time point. The absolute difference in the mean crude PTB rate between the most and least deprived quintiles increased by 35% from 9.1 per 1000 (6.7–11.5 per 1000) in 2012 to 12.3 per 1000 (9.2–15.4 per 1000) in 2017.

### Fixed-effects regression

Fixed-effects analysis revealed no significant association between unemployment and PTB (−0.39, *P*-value .089).

### Linear mixed-effects modelling

Model 1 (Table 1) estimated the effect of IMD quintile on the number of preterm live-and still-births per 1000 births in LAs. For LAs in the least deprived quintile, PTB rates were lower by 9.03 per 1000 births (95% CI −11.72: −6.34 per 1000) compared to those for LAs in the most deprived quintile. PTB rates were lower for LAs in IMD quintile four (by 6.60 per 1000 [95% CI −9.07: −4.13]) and quintile three (by 4.03 per 1000 [95% CI −6.4: −1.66]) when each was compared to the most deprived quintile. However, no difference in PTB rates was observed for LAs in quintile two compared to LAs in quintile one.

**Table 1:** Linear-mixed model findings for model 1 (‘Index of Multiple Deprivation’ as the predictor of interest).

Variables				Estimate	95% Confidence intervals	Type III ANOVA
Fixed effect	Exposure of interest	Index of Multiple Deprivation (IMD)	IMD 1 (most deprived)	Reference		
			IMD 2	−1.26	−3.63: 1.09	0.30
			IMD 3	−4.05	−6.46: −1.64	<0.01
			IMD 4	−6.64	−9.07: −4.13	<0.01
			IMD 5 (least deprived)	−9.1	−11.65: −6.55	<0.01
	Other covariates	Time		0.99	0.84: 1.14	<0.01
		Confounders	Maternal age: proportion of births in mothers aged > 35 years	−0.12	−0.26: 0.02	0.1
			Marital status: proportion or births outside marriage or civil partnership	0.08	−0.01: 0.17	0.1
Random effect: LA level residual variance	20.1					
No. of local authorities	295					
No. of observations	1663					

Model 2 (Table 2) analysed the association between trends in child poverty and trends in the crude PTB rate in LAs in England between years 2012 and 2016. We found that a 1 percentage point increase in the proportion of children in poverty was associated with an additional 0.24 PTBs per 1000 total births (95% CI 0.12: 0.36).

**Table 2:** Linear-mixed model findings for model 2 (‘child poverty’ as the predictor of interest).

Variables			Estimate	95% Confidence intervals		Type III ANOVA
Fixed effect	Exposure of interest	Child poverty		0.24	0.12: 0.36	<0.01
	Other covariates	Time		1.2	1.02: 1.38	<0.01
		Confounders	Maternal age: proportion of births in mothers aged > 35 years	−0.20	−0.34: −0.06	<0.01
			Marital status: proportion or births outside marriage or civil partnership	0.13	0.03: 0.23	<0.01
Random effect: LA level residual variance	16.5					
No. of local authorities	321					
No. of observations	1498					

Model 3 (Table 3) estimated the association between unemployment and the crude PTB rate in LAs between 2013 and 2017. No association between unemployment and PTB rates was identified during this period (estimate 0.14, 95% CI −0.11: 0.39).

**Table 3:** Linear-mixed model findings for model 3 (‘unemployment’ as the predictor of interest).

Variables				Estimate	95% Confidence intervals	Type III ANOVA
Fixed effect	Exposure of interest	Unemployment		0.14	−0.11: 0.39	0.28
	Other covariates	Time		1.15	0.91: 1.39	<0.01
		Confounders	Maternal age: proportion of births in mothers aged > 35 years	−0.23	−0.39: −0.07	<0.01
			Marital status: proportion or births outside marriage or civil partnership	0.20	0.10: 0.30	<0.01
Random effect: LA level residual variance	18.3					
No. of local authorities	321					
No. of observations	1605					

## Discussion

### Main findings of this study

There was a rising trend in PTB rates in England between 2012 and 2017. Although PTB rates increased for all deprivation quintiles during this period, rates positively correlated with increasing relative disadvantage. Mixed-effects modelling also revealed a significant deprivation gradient in PTB rates, with 9.03 fewer PTBs per 1000 births for the least deprived quintile when compared to the most deprived quintile.

Additionally, the absolute difference in the mean PTB crude rate for the most and least deprived quintiles increased by 35% from 2012 to 2017, suggesting a widening of PTB inequality. This observation echoes the findings of the ‘Health Equity in England: The Marmot Review 10 Years On’ review, which reported a concerning widening in health inequalities between 2010 and 2020.^24^

Our mixed-effects analysis demonstrated an association between child poverty and PTB; for each percentage point increase in child poverty, an estimated additional 0.24 per 1000 births was preterm. To contextualize our findings, between 2013 and 2023 the UK was observed to have the largest increase in relative child poverty out of 37 high-income countries, with an increase from 17.3% to 23.2% before housing costs.^25^ In 2024, there were ~ 570 000 total births in England.^26^ Although not directly comparable due to differing child poverty measures, applying the observed change in child poverty to the most recent year of births, we estimated that child poverty would have been associated with 816 PTBs. This is consistent with overwhelming evidence that socioeconomic position is a risk factor for PTB.^27^

Unemployment rates generally declined during the study period and were not found to be associated with PTB rates in English LAs. This may be a real observation and reflect the quality of employment and working conditions for the additional employees. UK benefit reforms over the past 25 years have incentivized benefit claimants to move into employment; however, this has largely been into part-time, low-paid work.^28^ Lower levels of job control and higher levels of job hazards and physical demands, commoner in manual occupations, are all associated with increased PTB risk.^29^ Alternatively, the absence of an association may be due to the ecological fallacy.

Fixed-effects and mixed-effects analyses showed that the affordability of home ownership was negatively associated with PTB rates (Supplementary Appendix). That is, as it becomes less affordable for residents in English LAs to purchase a house, there is an associated decline in PTB rates. This is inconsistent with existing research demonstrating socioeconomic disadvantage as a determinant of PTB. One potential explanation is ecological fallacy. For those whom poverty may be a significant driver of their PTB risk, the prospect of housing ownership may have been unlikely during this period. For instance, between 2012 and 2017, the ratio of an average-priced home to the number of years income equivalent remained relatively stable for lower-income households in England, at 11.65- and 12.58-times equivalent income, respectively.^30^ At this considerable scale, individuals affected by PTB increases may have been more likely to be renting^31^ and thus may not have been directly affected by affordability. However, as the mechanism for our finding remains inconclusive, this warrants further exploration.

### What is already known on this topic

There is strong evidence for socioeconomic disparities in PTB. A large systematic review found a significant association between socioeconomic measures and adverse birth outcomes (PTB, low birthweight, and/or small for gestational age) in 93 of 106 studies analysed.^27^ To our knowledge, no study has examined the effect of major SDH on recent PTB rates in England. However, child poverty has been shown to be a driver of other child health outcomes in England during the same period. Taylor-Robinson et al. (2019) assessed the impact of rising child poverty rates on rising infant mortality rates in England between 2014 and 2017. They identified that child poverty was associated with approximately one-third of excess infant deaths occurring during this time.^32^

### What this study adds

This study provides evidence to suggest that poverty was associated with the rising trend in PTB seen in England between 2012 and 2017. Understanding that areas with higher child poverty may experience higher rates of PTB is important, because it is projected that under existing policies an additional 1.5 million people will be living in relative poverty by 2029/30.^33^

Current national PTB guidelines for healthcare professionals do not consider SDH in preterm labour risk assessments. We recommend that this study is repeated using individual-level data to remove the effect of ecological fallacy. This will also facilitate identification of the dose–response thresholds at which SDH associated with living in poverty may pose a significant risk of PTB. This will allow SDH to be incorporated into clinical risk assessment tools for preterm labour, thereby increasing their sensitivity.

It is also imperative that national-level action is taken to tackle socioeconomic drivers of PTB. While causal inferences cannot be made about the association between poverty and PTB from our study alone, our study contributes to the wider causal narrative supported by extensive evidence.

### Limitations of this study

In the absence of individual-level data, we undertook an ecological study of area-level routine data. As such, it is susceptible to ecological fallacy. Further studies should examine the relationship between individual-level socioeconomic deprivation measures and PTB data.

Second, national-level trend data on PTB rates suggests that the rise in PTBs is being driven by the birth of moderately preterm babies. However, due to a low number of PTBs for some LAs, we were unable to conduct sub-group analysis due to the risk of statistical disclosure. By not limiting analysis to the moderately preterm gestational group, this may have resulted in an underestimation of SDH effect sizes.

Third, as discussed, there are concerns relating to the accuracy of the unofficial child poverty estimates utilized in this study; however, there is a lack of more robust alternate data sources for this time period.^23^

Fourth, studies have shown that maternal ethnicity is strongly associated with PTB risk.^11^ However, we could not examine this potential confounding effect due to the absence of an area-level indicator during the study period.

Fifth, between 7.9% and 24.1% of observations were missing from the LMMs (Table 1) due to incomplete routinely available datasets, which has the potential to introduce bias.

Sixth, IMD2019 was used as a time-invariant area-level measure of SES. This assumes that relative deprivation ranking remained stable over the period of interest, but it is unlikely to have remained static.

Additionally, to maintain uniform time intervals for data points across both exposures and outcomes, we considered each 3-year rolling crude PTB rate as an annual rate for the middle year within the period, thus there will be some overlap between consecutive ‘year’ outcome values.

Finally, the impact of child poverty on PTB may be even greater than estimated by this study due to how this indicator is constructed. It considers only those who receive out-of-work benefits or who receive tax credits and have an income < 60% of median income. It does not account for households who are eligible for tax credits or benefits but do not claim them. This could make a potentially significant difference as it is estimated that 33% and 19% of people eligible for Working Tax credit and Housing Benefit, respectively, do not claim them.^34^

## Conclusion

PTB is a leading cause of childhood death and morbidity as well as being a considerable economic burden. Rates of PTB increased in England for most of the last decade. It is vital that there are continued efforts to reduce PTB. Our analysis identified a deprivation gradient in PTB rates and a widening in PTB inequalities between the most and least deprived quintiles in England between 2012 and 2017. LMM showed a positive association between poverty and PTB. It is important that women living in poverty and areas of deprivation are recognized as having an increased risk of PTB during pre-conception or early pregnancy healthcare consultations, though further understanding is required to ensure that interventions are appropriately targeted. We therefore recommend further research of the association between living in poverty or in areas of socioeconomic deprivation and PTB risk, utilizing individual-level data. This should remove the threat of ecological fallacy and enable risk thresholds to be identified. This may aid in quantifying SDH-specific risk of PTB, allowing targeted, proportionate social support offers to be implemented for women facing differing levels of disadvantage, with an aim to reduce their PTB risk. Additionally, our findings provide further suggestion of the potential negative impact that rising poverty in England is having on the health of children. It is therefore vital that immediate action is taken by the UK government to introduce stronger poverty-reduction policies.

## Supplementary Material

Research_article_SDH_and_PTB_Appendix_fdag045

## Data Availability

Data are available upon reasonable request.

## Patient and public involvement

This study is part of a collective of research informed by public insights gathered during discussions surrounding the UNICEF Child Friendly City programme in Liverpool, UK. Children and families have endorsed research which concerns social inequalities in children and young people’s health. The findings of this programme of work contribute to our continuing consultations with young people and service providers about the importance of addressing child health inequalities.

## Copyright

Copyright is assigned to The Faculty of Public Health.

## Notes

**Amy Wilcock**, Public Health Specialty Registrar

**Davara Bennett**, NIHR SPHR Postdoctoral Launching Fellow

**David Taylor-Robinson**, W.H. Duncan Chair in Health Inequalities, Professor of Public Health and Policy, NIHR Research Professor, Honorary Consultant in Child Public Health at Alder Hey Children's Hospital

**Philip McHale**, Senior Clinical Lecturer
