# Supplementary material for: Assessing the impact of social determinants of health on rising preterm birth trends in England. A longitudinal ecological study, 2012–17
Source: J Public Health (Oxf). 2026 Jun 16;48(3):780–7. doi: 10.1093/pubmed/fdag045 (PMC13531721; doi:10.1093/pubmed/fdag045)
Supplement: Supplementary file 1 [file research_article_sdh_and_ptb_appendix_fdag045.docx]

Appendix

**Missing Data**

Table 4 provides a breakdown of missing data in our study. In model 2, 329 of the 476 observations missing resulted from all 329 LAs having no data available for the year 2017. Similarly, in model 3, 329 of the 369 observations missing resulted from all 329 LAs having no data available for the year 2012.

| Data included in each LMM | Model 1 | Model 2 | Model 3 | Model 4 |
| --- | --- | --- | --- | --- |
| Number of LAs included in the models  (out of a maximum of 329 LAs) | **295** | **321** | **321** | **321** |
| Number of LAs missing from the models  (i.e. LAs for which there is no data at all for the period of 2012-2017) | **34** | **8** | **8** | **8** |
| Number of observations  (out of a maximum of 1974 observations, assuming there are observations for all LAs for each year within the 2012-2017 period) | **1663** | **1498** | **1605** | **1819** |
| Number of observations missing from the models | **311** | **476** | **369** | **155** |
| Percentage of observations missing from the models (%) | **15.8** | **24.1** | **18.7** | **7.9** |

Table 4: Data missing from the LMMs

**Further Statistical Analysis: Examining trends in child poverty and unemployment by local authority**

We analysed trends in child poverty and unemployment by LA using spaghetti plots (Figure 2 and Figure 3 respectively). The plots demonstrated limited within-LA variation in both child poverty (between 2012-2016) and unemployment (between 2013-2017).

Figure 2: A spaghetti plot of trends in child poverty by local authority

Figure 3: A spaghetti plot of trends in unemployment by local authority

**Assumption Testing for the Linear mixed effects modelling**

Assumption testing for the linear mixed effects modelling demonstrated that the residuals were normally distributed. Residuals versus fitted plots revealed homoscedasticity of the residuals. Additionally, the linearity assumption was met. VIF values were between 1 and 3 for all variables across the models, representing a low strength of correlation between variables within models and thus no multicollinearity.

**Further Statistical Analysis: Affordability of home ownership**

**Linear Mixed Model**

We also estimated the correlation between trends in affordability of home ownership (ratio of house price to annual residence-based earnings) and PTB crude rates between 2012 and 2017 (Table 5). A total of 1819 observations from 321 LAs were examined. The model estimated that, for LAs in England between 2012 and 2017, when adjusted for maternal age and marital status, an increase in the ratio by 1 (increase in home unaffordability) was associated with a decrease in the PTB rate of 0.29 preterm births per 1,000 births (95% CI -0.56: -0.02). Similarly, fixed effects analysis (Table 6) for this predictor showed that home unaffordability was associated with a decrease in the PTB rate of 0.49 per 1,000 births (p= 0.036).

| Variable | | | | Estimate | 95% Confidence Intervals | Type III ANOVA |
| --- | --- | --- | --- | --- | --- | --- |
| Fixed effect | Exposure of interest | Home Unaffordability | | -0.29 | -0.56: -0.02 | <0.05 |
|  | Other Covariates | Time | | 1.12 | 0.94: 1.30 | <0.01 |
|  |  | Confounders | Maternal Age: Proportion of births in mothers aged >35 years | -0.16 | -0.32: -0.003 | <0.05 |
|  |  |  | Marital status: Proportion or births outside marriage or civil partnership | 0.18 | 0.08: 0.28 | <0.01 |
| Random effect: LA level  Residual variance | 18.7 | |  |  |  |  |
| No. of local authorities | 321 | |  |  |  |  |
| No. of observations | 1819 | |  |  |  |  |

Table 5: Linear mixed model findings for model 4 (‘Home Unaffordability’ as the predictor of interest).

| Fixed effects models | Estimate | p-value |
| --- | --- | --- |
| Home Unaffordability | -0.49 | 0.036 |

**Fixed effects model: Affordability of Home Ownership**

Table 6: Fixed effects analysis with 'home unaffordability' as the predictor of interest
